# Pediatric inflammatory bowel disease: Is it really uncommon in Asian children?

**DOI:** 10.1002/jgh3.12330

**Published:** 2020-04-26

**Authors:** Ujjal Poddar, Surender Kumar Yachha, Anshu Srivastava, Niraj Kumari

**Affiliations:** ^1^ Department of Pediatric Gastroenterology Sanjay Gandhi Postgraduate Institute of Medical Sciences Lucknow India; ^2^ Department of Pathology Sanjay Gandhi Postgraduate Institute of Medical Sciences Lucknow India

**Keywords:** antitubercular treatment trial, incidence, progression

## Abstract

**Background/Aim:**

Inflammatory bowel disease (IBD) is said to be rare in Asian children, and there is scarce information from India. We therefore analyzed our experience of pediatric IBD.

**Methods:**

Prospectively maintained data of 105 consecutive children [median age 12 (IQR:7–14) years, 71 males] with IBD from July 2001 through June 2016 were retrospectively analyzed. Their detailed clinical features, endoscopic appearance, histopathology, and treatment outcomes were recorded. For Crohn's disease (CD), disease phenotype and disease location were assessed as per Paris classification.

**Results:**

Disease spectrum includes ulcerative colitis (UC), 55 (52%); CD, 43 (41%); and IBD‐unclassified, 7 (7%). There was a significant increase in the number of cases in the last 5 years compared to the previous 10 years (63 *vs*. 42, r^2^ = 0.96). Most UC cases (75%) had extensive/pancolitis, 74% of CD had colonic/ileocolonic disease, and 65% had inflammatory phenotype. Fever, growth failure, pain in abdomen, and need for surgery were significantly more frequent in CD than in UC (*P* < 0.0001). Over a median follow up of 19 (IQR: 7–48) months, remission was achieved in 48 of 51 (94%) UC patients and in 24 of 34 (70.6%) CD patients; an immunomodulator was required to maintain remission in 67% of UC cases. In CD, there was a significant reduction in the use of empirical antitubercular therapy (76%, *P* = 0.008) with time, and disease progressed in three.

**Conclusions:**

IBD is not uncommon, and the incidence seems to be increasing among Indian children. UC is more common than CD and is more often an extensive disease. CD is mainly an inflammatory phenotype. The majority of children with IBD required an immunomodulator to maintain remission.

## Graphical abstract

Inflammatory bowel disease (IBD) is said to be rare in Asian children, and we analyze our experience of 105 children: UC 52%, CD 41%, and IBD‐U 7%. There was an increasing incidence of IBD over the years. In CD, there was a significant reduction in the use of empirical antitubercular therapy with time (76%, *P* = 0.008) prior to starting immunosuppressant therapy.

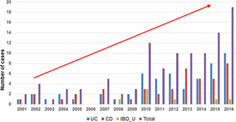

## Introduction

Inflammatory bowel disease (IBD) is relatively less common in children compared to adults. Around a quarter of all IBD cases present in the first two decades of life.^1^ In contrast to adult‐onset IBD, Crohn's disease (CD) is almost twice as common as ulcerative colitis (UC) in children (pediatric IBD).^2^, ^3^ In India, there seems to be a regional difference in the prevalence of CD vis‐à‐vis UC. In North India, UC is more common than CD, whereas in South India, CD is more common than UC.^4^ There is an increasing incidence of IBD, especially CD, both in adults and in children all over the world.^5^ However, the incidence and the prevalence rates of IBD in Asia are much lower than that in Europe or North America, and it is believed that IBD is uncommon in Asian children.^6^ There is scarce information about pediatric IBD from India except a case series of 10 children with CD^7^ and a brief report of 34 cases of pediatric IBD from South India.^8^

In developing countries, as also in India, infective causes such as tuberculosis are always considered first over CD, and physicians are reluctant to start immunosuppressive/immunomodulator drugs without giving antitubercular therapy (ATT) a fair trial.^9^ There is a subjective impression that, with increasing awareness about CD, this practice of ATT trial prior to immunosuppressive therapy is decreasing. Nevertheless, there are no data yet to support this notion.

We therefore analyzed our experience of IBD in children seen in a tertiary care referral hospital of North India over the last 15 years to study the clinical spectrum of pediatric IBD in India, the change in the number of cases diagnosed over the years, and if the practice of an ATT trial before starting immunosuppressant/immunomodulator in CD is indeed decreasing.

## Methods

Prospectively maintained data of 105 consecutive children with IBD were retrospectively analyzed in this study. Consecutive children (age: 18 years or less) diagnosed with IBD from July 2001 through June 2016 were included in this study. Follow‐up data were collected till December 2016. In our department, we follow a protocol‐based approach to diagnose and manage IBD cases. All children suspected to have IBD, irrespective of its type (UC, CD, or IBD‐unclassified), undergo full colonoscopy with ileal intubation, upper gastrointestinal endoscopy with protocol biopsy (from duodenum, antrum and body of stomach, and esophagus), and small bowel evaluation. Small bowel evaluation was carried out with a barium meal follow‐through in the first 10 years of the study and magnetic resonance (MR) enterography or CT enterography in the latter 5 years. The diagnosis of IBD was made as per European Society of Pediatric Gastroenterology, Hepatology and Nutrition (ESPGHAN) revised Porto criteria^10^ and classified as per Paris classification.^11^ IBD was classified as UC, CD, and IBD‐unclassified (IBD‐U). In all cases of suspected CD, tuberculosis was ruled out using the tuberculin skin test (TST), chest x‐ray, gastric aspirate for acid‐fast bacilli (AFB), and tissue specimen such as intestinal biopsy for Mycobacterium Tuberculosis polymerase chain reaction (TB‐PCR) and Ziehl‐Neelsen stain for acid‐fast bacilli staining.

UC was diagnosed when there was diffuse and continuous macroscopic inflammation beginning in the rectum and extending proximally to a variable extent and inflammation limited to mucosa microscopically with normal small bowel (except features of backwash ileitis such as hyperemia, edema, superficial ulcers) on ileocolonoscopy.^12^ CD was suspected on endoscopy when there was discontinuous inflammation with intervening zones of normal bowel, fissuring ulceration (deep, serpiginous, longitudinal), stricture and fistula formation, and cross‐sectional imaging and/or histopathology showing transmural disease with/without granulomas. In addition, the diagnosis of CD was strongly considered when there was small bowel pathology on imaging, such as transmural thickening of bowel loops with or without ulcers.^12^ Attempts were made to obtain tissue diagnosis for CD (transmural inflammation with or without noncaseating granuloma away from the site of ruptured crypts). Video capsule endoscopy (VCE) and balloon‐assisted enteroscopy were not performed regularly. A suspected case of colonic IBD with macroscopic continuous disease in a setting of microscopic discontinuous disease or inflammation extending beyond muscularis mucosa was labeled as IBD‐U.^12^ The severity of UC was assessed by using modified Truelove and Witt's Criteria.^13^

As per revised Porto guidelines^10^ the diagnostic workup of a suspected case of IBD includes: ileocolonoscopy, upper gastrointestinal endoscopy, and small bowel evaluation in all except typical cases of UC. In our cohort, of 50 cases of CD and IBD‐U (43 and 7 cases, respectively), all three investigations (upper and lower gastrointestinal endoscopy and small bowel evaluation) were performed in all but two cases (CD was diagnosed on the basis of CT scan followed by laparotomy in them). Among the UC cases (*n* = 55), upper gastrointestinal (UGI) endoscopy and small bowel evaluation along with colonoscopy were performed in 34 (62%) cases, both upper and lower GI endoscopy in 11 (20%) cases, and the remaining 10 (18%) were diagnosed on the basis of colonoscopy alone. Ileal intubation was successful in 84 of 100 cases (84%); colonoscopy was not done in 2; and in 3, limited colonoscopy was performed (in the first 2 years of our study).

All cases of UC, except those with mild disease, were treated with corticosteroids (prednisolone, 2 mg/kg, maximum 40 mg/day) through induction therapy for 2–4 weeks and were then tapered off over 2–3 months at the rate of 5 mg/week. They were subsequently maintained on 5‐aminosalicylate (5‐ASA) (40‐60 mg/kg/day). An immunomodulator (azathioprine at a dose of 2 mg/kg/day) was added if there was a relapse while a patient was on maintenance 5‐ASA. Total colectomy was performed in cases with acute severe colitis and those unresponsive to intravenous steroids and antibiotics for 7 days. All cases of CD were treated with an induction regimen of corticosteroids along with azathioprine (with 5‐ASA for colonic CD). Maintenance therapy included azathioprine with/without 5‐ASA. In CD, bowel resection was performed when there was ongoing obstruction despite medical therapy or complications such as perforation, acute bleeding, or internal fistula.

### *Ethical statement*

This study conformed with the Helsinki Declaration of 1975, as revised in 2000 and 2008, concerning human and animal rights, and the policy concerning informed consent was followed in this study. This study was carried out after obtaining due ethical clearance from the Institute Ethics Committee, and informed consent was obtained from either parent before all procedures.

### *Statistics*

Data were analyzed using SPSS version 15 (SPSS, Inc., Chicago, IL, USA). Discrete variables are presented as number with percentage and continuous variables as median with interquartile range (IQR). A comparison was made using Chi‐square or Fisher's exact test for discrete variables and student's *t*‐test for continuous variables. Binary logistic regression analysis was used for multivariate analysis to find the features that differentiate CD from UC. A value <0.05 was considered significant.

## Results

**Figure 1:**
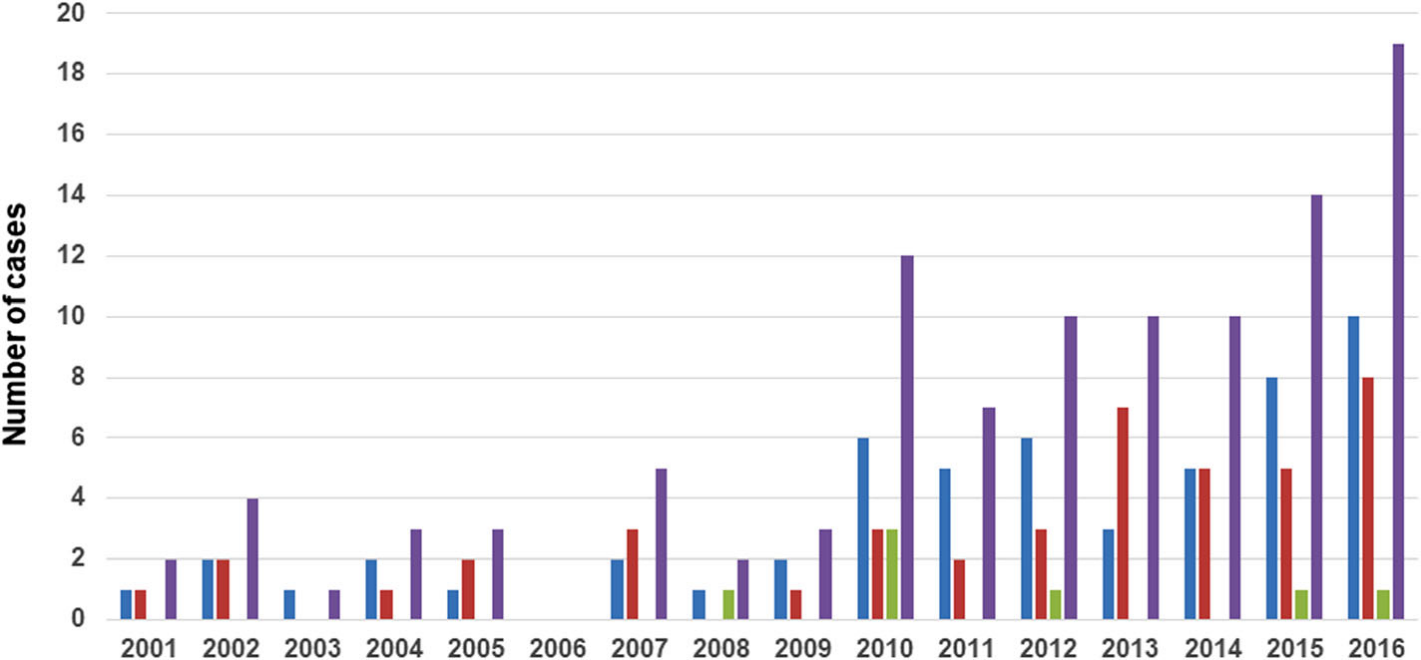
Bar diagram showing year‐wise distribution of cases (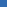, UC; 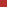, CD; 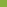, IBD‐U; and 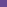, total IBD).

**Figure 2:**
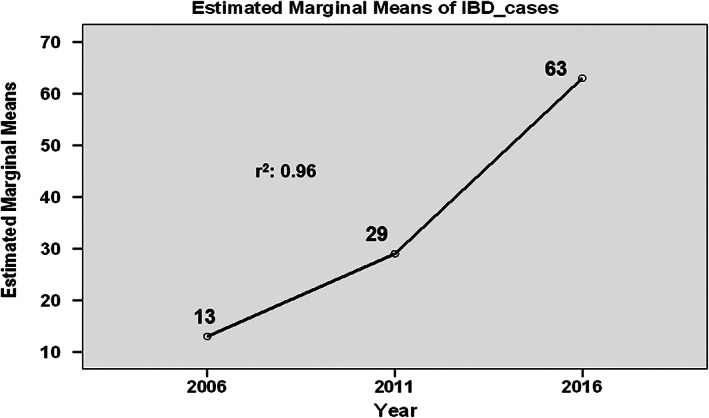
Linear regression analysis showing increasing number of IBD cases diagnosed in our center over the years (2001–2016): every 5 years.

**Figure 3:**
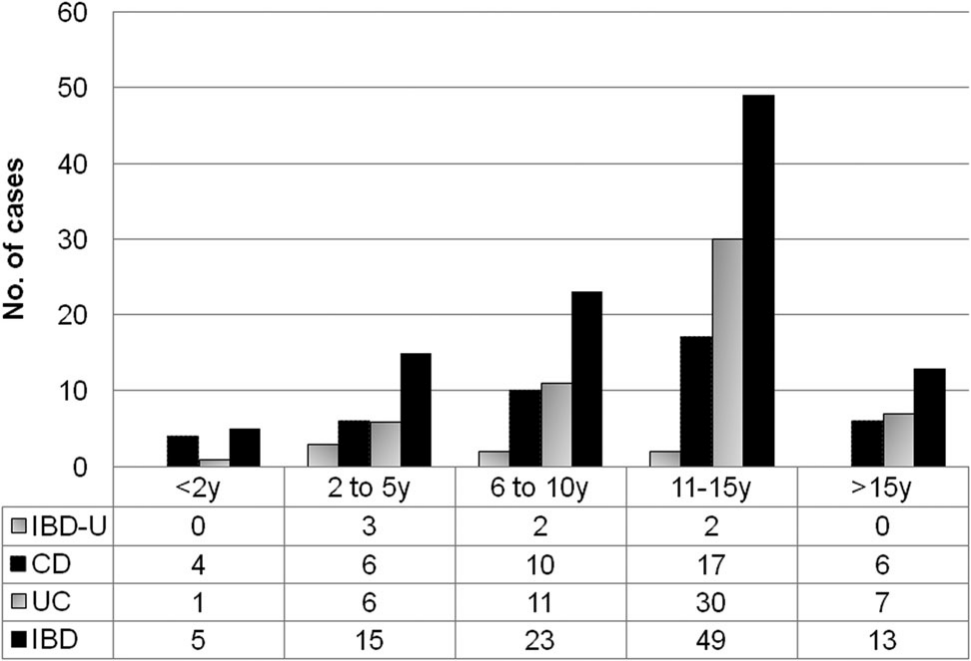
Bar diagram showing age‐wise distribution of cases.

IBD was diagnosed in a total of 105 cases during the study period. The median age was 12 (IQR 7–14) years, with a male‐to‐female ratio of 71: 34. UC was diagnosed in 55 (52%), CD in 43 (41%), and IBD‐U in 7 (7%). The year‐wise distribution of cases (UC, CD, and IBD‐U) is shown in Figure 1. On linear regression analysis, there was a significant increase in the number of cases in the last 5 years compared to the previous 10 years (63 *vs* 42, *r* ^2^ = 0.96), with no change in the proportion of UC *versus* CD (Fig. 2). Age‐wise distribution of cases is shown in Figure 3. A majority of the cases (69%) were in the 6–15 years age group. Very early‐onset (<6 years of age) IBD was seen in 20 (19%) cases, and 5 of them were diagnosed in the first 2 years of life (infantile IBD). The youngest child was a 9‐month‐old girl with CD.

Clinical features of UC and CD are shown in Table 1. Most children (41, 75%) with UC had extensive/pancolitis, 10 (18%) had left‐sided UC, and 4 (7%) had ulcerative proctitis. Almost half of the UC cases (27, 49%) presented with moderately severe disease; 20 (36.5%) had severe disease, and 8 (14.5%) had mild disease. There was significant correlation between extent of the disease and severity of illness (*P* = 0.01). All severe cases had extensive/pancolitis (extensive/pancolitis *vs* others, *P* = 0.003), and all mild cases had either left‐sided colitis (50%) or ulcerative proctitis (50%) (Table 2).

**Table 1:** Comparisons between ulcerative colitis (UC) and Crohn's disease (CD)

Clinical features	UC (*n* = 55)	CD (*n* = 43)	*P* (Univariate)
Median age (IQR)	12 (8–14)	12 (6–14)	0.23
Male: female	37:18	29:14	1.00
Duration of symptoms (months)	18.20 ± 14.41	18.85 ± 15.33	0.83
Weight *z* score	−1.88 ± 1.03	−2.82 ± 1.28	0.0002
Height *z* score	−1.36 ± 1.21	−2.14 ± 1.55	0.007
Pain abdomen (%)§	14 (25.4%)	30 (69.7%)	0.0001
Diarrhea (%)	55 (100%)	31 (72%)	0.0001
Rectal bleeding (%)	55 (100%)	27 (63%)	0.0001
Weight loss (%)§	27 (49.1%)	40 (93.0%)	0.0001
Anorexia (%)	16 (29.1%)	36 (83.7%)	0.0001
Fever (%)§	8 (14.5%)	22 (51.1%)	0.0001
Need for surgery (%)	3 (5.4%)	17 (39.5%)	0.0001
Extraintestinal manifestations (%)	11 (20.0%)†	10 (23.2%)‡	0.805
Perianal disease (%)	0	6 (13.9%)	0.005

† Aphthous stomatitis 4, arthritis 4, deep vein thrombosis 2, aphthous stomatitis and arthritis 1 each.

‡ Aphthous stomatitis 5, arthritis 3, aphthous stomatitis and arthritis 1 each, uveitis with oral and joint 1.

§ *Multivariate analysis (binary logistic regression)*: Pain in abdomen (*P* = 0.02), weight loss (*P* = 0.009), and fever (*P* = 0.03) were significant.

**Table 2:** Ulcerative colitis: Severity and extent cross‐tabulation (*n* = 55)

		Extent of the disease			
		Extensive/pancolitis	Left‐sided ulcerative colitis	Ulcerative proctitis	Total
Severity	Severe (count, % within severity)	20 (100.0%)†	0 (0.0%)	0 (0.0%)	20 (100.0%)
	Moderately severe (count, % within severity)	21 (77.8%)	6 (22.2%)	0 (0.0%)	27 (100.0%)
	Mild (count, % within severity)	0 (0.0%)	4 (50.0%)	4 (50.0%)	8 (100.0%)
Total	(count, % within severity)	41 (74.5%)	10 (18.2%)	4 (7.3%)	55 (100.0%)

† Pearson Chi‐Square: 39.573, *P* = 0.000.

Disease location and phenotypes of CD cases are shown in Table 3. The majority of children (32, 74%) with CD had colonic or ileocolonic disease. Additional upper gastrointestinal (UGI) involvement (L4) was seen in four cases (two with L3, one each with L1 and L2). Of 43 CD patients, 28 (65%) had inflammatory or nonstricturing nonpenetrating (B1) phenotype. On histopathology, 13 of 43 (30%) patients had ill‐formed granulomas (nine from colonic biopsy, two from resected small bowel, one on upper GI biopsy, and one from mesenteric lymph node). With increasing awareness and confidence in the diagnosis of CD, empirical antitubercular therapy (trial for 6–9 months) prior to initiating immunosuppressant/immunomodulator therapy has reduced significantly in the last 5 years [4 of 4 (100%) in the first 5 years, 4 of 10 (40%) in the second 5 years, 7 of 29 (24%) in the last 5 years; *P* = 0.045 between the first 10 years (8/14, 57%) and the last 5 years (7/29, 24%); *P* = 0.008 between the first and last 5 years]. The comparisons between UC and CD are given in Table 1. Clinical features that discriminate CD from UC (on multivariate analysis) were pain in abdomen, weight loss, and fever.

**Table 3:** Comparisons of disease location and phenotype in Crohn's disease

		Vernier‐Massouille *et al*.^36^ (*n* = 281)	Van Limbergen *et al*.^34^ (*n* = 273)	de Bie *et al*.^37^ EUROKIDS Registry (*n* = 582)	Buderus *et al*.^38^ CEDATA‐GPGE Registry (*n* = 480)	Present study (*n* = 43)
Disease location†	L1	39 (14%)	16 (5.9%)	95 (16%)	64 (13.3%)	11 (26%)
	L2	47 (17%)	99 (36.3%)	159 (27%)	95 (19.8%)	16 (37%)
	L3	194 (69%)	138 (50.5%)	307 (53%)	300 (62.5%)	16 (37%)
	L4‡	102 (36%)	139 (50.9%)	172 (30%)	257 (53.5%)	4 (9%)
Disease phenotype§	B1	71%	249 (91.2%)	959/1177¶ (82%)	95.1%	28 (65%)
	B2	25%	12 (4.4%)	144/1177 (12%)	2.8%	11 (26%)
	B3	4%	12 (4.4%)	55 (5%) B2B3:19 (2%)	2.1%	4 (9%)

† L1: ileal, L2: colonic, L3: ileocolonic, L4: upper gastrointestinal involvement.

‡ L4 disease in addition to L1/L2 or L3.

§ B1: inflammatory, B2: stricturing, B3: penetrating.

¶ Information on disease behavior was available in 1177 cases.

In the UC group, the treatment was started with oral corticosteroids with sulfasalazine in 49 patients and sulfasalazine alone in 6 patients. Of the four cases with acute severe colitis, one responded to cyclosporine, and three required colectomy. Over a median follow up of 19 (IQR: 7–48) months, remission was achieved with steroids in 48 (87%) patients; 3 cases of acute severe colitis did not respond to steroids and required surgery, and 4 were lost to follow up. On follow up, one or more relapses occurred in 30 of 48 (62.5%) of those who achieved remission, and an immunomodulator (azathioprine) was required to maintain remission in 32 of 48 (67%) cases. One child developed frequent relapses despite being on azathioprine and was switched to a biological (infliximab), and he is doing well. One child on azathioprine developed cytopenia and was switched to methotrexate.

In the CD group, one child (with B3 phenotype) died before starting therapy, 8 (phenotype B1: 5, B2: 2, and B3:1) were lost to follow up after starting initial therapy, and the remaining 34 were on azathioprine after initial steroids. Over a median follow up of 16 (IQR: 5.5–46.5) months, remission was achieved in 24 (70.6%) cases [in 18 with medical therapy alone and in 6 after surgical resection]. Remission could not be achieved in 10 (29%) despite surgery and biologicals. Biologicals were used in seven children (infliximab six, adalimumab one). After three doses of induction therapy with infliximab, two children went on to remission; remission was maintained with azathioprine in one, and the other child relapsed on two maintenance doses of infliximab. There was no response in the remaining four after induction therapy. One child on adalimumab showed response and is on maintenance adalimumab. On follow up, the disease progressed in three (B1 to B2 in 2, B1 to B3 in 1). Overall, 17 (50%) cases required surgery, small bowel resection was performed in 12 (9 for obstruction and 3 for bleeding), subtotal colectomy in 3 for bleeding (with terminal ileal resection in two), right hemicolectomy in 1 (for cecal perforation during colonoscopy), and ileostomy in an infantile case of IBD who was refractory to biologicals. The median age of the seven IBD‐U cases was 8 (IQR: 3.5–11.5) years, with a male‐to‐female ratio of 5:2. Over a median follow up of 45 (IQR: 15–49) months, two of the seven (28.6%) IBD‐U children were reclassified as CD, and the remaining five remained in the IBD‐U group. One child with infantile IBD who had multiple internal and external fistulas and was refractory to biologicals was found to have IL‐10 receptor polymorphism, and the child is planned for hematopoietic stem cell transplant (HSCT).

## Discussion

This is the largest single‐center cohort study on pediatric IBD from India. In this hospital‐based study, we have shown the increasing number of cases diagnosed with IBD in our center over the years, and in North India, similar to adults, UC is more common than CD. UC is predominantly a more extensive disease, and CD is mainly a colonic or ileocolonic disease with nonstricturing nonpenetrating phenotype. Most children, both with UC and CD, required an immunomodulator to maintain remission, and a third of CD cases failed to achieve remission despite biologicals and surgical resection.

In pediatric IBD, CD is seen more frequently than UC. However, for some unknown reason, UC is reported to be more frequent than CD in North India. This has been shown in adult and pediatric studies.^4^, ^13^ On the contrary, the incidence of CD is almost twice that of UC in South India. A nation‐wide questionnaire‐based prospective survey among gastroenterologists reported 1159 cases of IBD in adults from India.^4^ The ratio between UC and CD was 2:1 in North India and 1:1 in South India.^4^ Another questionnaire survey of 221 cases of pediatric IBD from India has documented the ratio between UC and CD of 1.8:1 in North India and 1:2 in South India.^14^ Our center is situated in North India, and we have also diagnosed more UC than CD (1.3:1), but the ratio in favor of UC is not as large as has been reported earlier.^4^, ^14^ This difference in distribution of UC and CD may be related to the differences in genetic heterogeneity, hygiene, and diet between the people of North and South India.^15^, ^16^ In our study as well, we found more UC cases than CD, and the proportion remained the same despite an increase in incidence in overall IBD. A recent study on 95 605 incident cases of CD and 112 004 incident cases of UC showed that the age of IBD onset varies with gender. They showed that female patients have a lower risk of CD during childhood, until the age range of 10–14 years, after which they had a higher risk of CD. They suggested that, with epigenetic and genetic risk factors, sex hormones could affect the pathogenesis of IBD.^17^ Disproportionately high male patients in our study may be due to gender discrimination in seeking medical advice, which is frequent in this part of the world. Clinical features and the differences between UC and CD noted in our study were the same as reported by Kugathasan *et al*. from the United States^18^ and Sawczenko *et al*.^19^ from the United Kingdom.

In a systematic review of 140 studies from 38 countries, Sykora *et al*.^20^ showed that the highest annual incidence of pediatric IBD is in Europe (23 per 100 000 person‐year), followed by North America (15.2 per 100 000 person‐year), and it is the lowest in Asia/the Middle East and Oceania (11.4 per 100 000 person‐year). In fact, barring a small study of 32 cases from Singapore,^21^ all other studies from Asia^22^, ^23^, ^24^, ^25^ have shown a low incidence of pediatric IBD (0.5–5.5 per 100 000 person‐year). They also showed a steady increase in the incidence of IBD over time globally. Increasing incidence has recently been reported from other Asian countries such as Japan,^26^ Korea,^27^ and Singapore.^28^ Although the present study is not a population‐based study to address the true incidence of pediatric IBD in the community, the increasing number of cases diagnosed in our center may be due to the true increase in incidence in the community or may be due to referral bias as ours is the only tertiary care referral center in North India.

Pediatric IBD consortium data of 1370 cases from the United States showed that the age of diagnosis was between 6 and 12 years in 48% of cases and 13 and 17 years in 37% of cases, while only 6% were 0–2 years old, and another 9% were in the 3–5 years age group.^29^ The mean age of 2087 children with IBD in the EUROKIDS registry data was 12.1 years (range, 0.6–17.9 years); however, a majority of the cases were between 7 and 17 years old.^30^ The median age of 739 new IBD cases from the United Kingdom^19^ was 12.6 years (range, 0.35–16); 4% were younger than 5 years old and 17% were between 5 and 10 years old. In our study as well, most children (69%) were in the 6–15 years age group, and we had only five (4.8%) cases in the 0–2 years age group (infantile IBD). Infantile IBD (onset in first two years of life)^10^ is altogether a different disease with strong genetic influence. In a study of 14 cases of infantile IBD from Korea, half of them had a mutation in the IL‐10 signaling pathway.^31^ This subset of patients has a more severe form of the disease, including perianal fistulas, and they do not respond to conventional medical therapy. The only cure for them is allogenic HSCT.^32^, ^33^

Compared with adults, UC in children is usually more extensive and more severe, and by 10 years after diagnosis, the colectomy rate is almost double (40% *vs* 20%, respectively).^3^, ^34^ In a study of 416 patients with pediatric IBD (276 CD, 99 UC, 41 IBD‐U) and 1297 cases with adult‐onset IBD, Van Limbergen *et al*.^34^ showed that 74.7% of UC had extensive colitis at presentation *versus* 48% of adults, and at 10 years of follow up, 40.9% of pediatric UC required colectomy *versus* 19.9% of adult‐onset UC. An analysis of 643 cases of UC in the EUROKIDS registry, collected from 2004 to 2009, showed extensive or pancolitis in 77% of cases, left‐sided colitis in 18%, and proctitis in 5% of cases.^35^ In our study, 75% had extensive/pancolitis at presentation, and 85% presented with moderate to severe disease. However, the colectomy rate was much lower in our study (5.4%). Although follow up was relatively short (median 19 months) in our study, all three children required colectomy at presentation (acute severe colitis) and none on follow up.

Pediatric CD behaves differently than that of adult‐onset CD. The majority of pediatric CD cases have ileocolonic (L3) or colonic (L2) disease, whereas small bowel CD (L3) dominates adult‐onset CD.^3^ Inflammatory phenotype (B1) dominates pediatric CD, while stricturing (B2) and penetrating (B3) phenotypes are common in adult‐onset CD.^3^ The comparisons between our study and that from France^36^ of 281 cases of CD in children, 273 cases of pediatric CD from the United Kingdom,^34^ 582 cases from the EUROKIDS registry,^37^ and 480 cases from the CEDATA‐GPGE registry^38^ from Germany are shown in Table 3. Results of the present study are almost similar, with the predominant location being ileocolonic in 74% of cases. However, the proportion of cases with ileal disease (L1) is higher (26%) in our study. Another observation is the involvement of the upper gastrointestinal tract (L4), which is much less in CD (9%) in our study than the reported figure of 30–53%.^34^, ^36^, ^37^, ^38^ This may be due to a smaller number of cases of CD in our study population. Disease behavior is almost similar in our study with a predominant inflammatory phenotype (65%); however, the proportion of stricturing phenotype (B2) is higher than the UK study,^34^ EUROKIDS registry.^37^ and CDATA‐GPGE registry.^38^ CD in children progresses from inflammatory to stricturing and penetrating phenotype on follow up. Vernier‐Massouille *et al*.^36^ showed that, over a follow‐up period of 10 years, B2 and B3 phenotypes increased from 25 to 44%, whereas the B1 phenotype decreased from 71 to 41%. Limbergen *et al*.^34^ showed that, even with shorter follow up of 4 years, there was a significant progression of disease (B2 plus B3 increased from 8.8 to 24.3% and B1 decreased from 91.2 to 75.8%, *P* < 0.001). Although our follow up was short (median 16 months), we have documented the progression of disease in 3 of 34 (9%) cases. The proportion of CD cases that required surgery was higher in our study than the reported figure of 17.1% by Limbergen *et al*.^34^ but was similar to that reported in adults (52.8%).^34^ This may be because we had a relatively higher proportion of L1 disease, and it is substantiated by the fact that 12 of 17 surgeries were small bowel resection.

In a recent study of 3461 cases of pediatric IBD from the EUROKIDS registry, Winter *et al*.^39^ showed that the proportion of IBD‐U has decreased from 7.7 to 5.6% on follow up (median 5.7 years) after complete investigation (52% had incomplete investigation, mainly due to lack of small bowel evaluation, to start with). After reinvestigation, the diagnosis of IBD‐U was changed to CD in 12% and to UC in 20% of cases. Pediatric IBD consortium registry data showed that 179 (13%) of 1370 cases of pediatric IBD had IBD‐U at presentation, and on follow up, 27 (15%) of 179 IBD‐U were reclassified to CD and 11 (6%) to UC.^29^ Paul *et al*.,^40^ from Bristol, followed up 25 IBD‐U cases for 4.5–11.5 years. On endoscopic re‐evaluation, the diagnosis was changed to CD in 7 (28%) and to UC in 3 cases (12%). As we are following a protocol‐based approach in all cases of IBD, the proportion of IBD‐U is only 7% in our series, and on follow up, diagnosis was revised to CD in two (28%) cases. Our data substantiate the recent observation of Winter *et al*.^39^ that complete workup, including small bowel evaluation, reduces the number of cases with IBD‐U, and regular follow up with repeat assessment, as and when required, changes the diagnosis in a quarter of cases.^41^

Intestinal tuberculosis is rampant in developing countries such as India, and it is a great mimic of CD. Sometimes clinically, endoscopically, radiologically, and even histologically, it may be difficult to distinguish CD from intestinal TB.^42^ In such situations, empirical ATT is attempted.^9^ However, with increasing awareness about features that help in differentiating CD from TB and the availability of better tests for microbiological confirmation of intestinal TB [such as TB‐PCR, cartridge‐based nucleic acid amplification test (CB‐NAT or geneXpert)], the empirical use of ATT before starting immunosuppressive therapy in CD should decrease, but no study confirms this notion. Our study has clearly shown that, with time, use of empirical ATT has decreased significantly (from 100 to 24%).

## Conclusions

IBD is not so uncommon in Indian children, and the incidence seems to be increasing in recent years. However, unlike in Europe, UC remains more common than CD in North India and is more often an extensive disease. CD is mainly a nonstricturing nonpenetrating disease in children and is predominantly of an ileocolonic or colonic type. The majority of children with IBD required an immunomodulator to maintain remission, and despite using biologicals and surgery, remission could not be achieved in a third of CD cases. With increasing awareness, the use of empirical ATT has decreased significantly in CD cases from 100% in the first 5 years to just 24% in the last 5 years of the study. IBD‐U should be reinvestigated as and when required as diagnosis changes in a quarter of cases.
